# Age targeting and scale-up of voluntary medical male circumcision in Mozambique

**DOI:** 10.1371/journal.pone.0211958

**Published:** 2019-02-22

**Authors:** Juan Dent, Nuno Gaspar, Emmanuel Njeuhmeli, Katharine Kripke

**Affiliations:** 1 The Palladium Group, Washington DC, United States of America; 2 USAID/Mozambique, Maputo, Mozambique; 3 USAID/Eswatini, Mbabane, Eswatini; 4 Avenir Health, Washington DC, United States of America; Vanderbilt University Medical Center, UNITED STATES

## Abstract

**Background:**

The voluntary medical male circumcision (VMMC) program in Mozambique aimed to increase male circumcision (MC) coverage to 80 percent among males ages 10 to 49 by 2018. Given the difficulty in attracting adult men over age 20 for circumcision, Mozambique became interested in assessing its age-targeting strategy and progress at the provincial level to inform program planning.

**Methods:**

We examined the impact and cost-effectiveness of circumcising different age groups of men using the Decision Makers’ Program Planning Toolkit, Version 2.1 (DMPPT 2). We also applied the model to assess the scale-up efforts through the end of September 2017 and project their impact on HIV incidence through 2030. The DMPPT 2 is a compartmental Excel-based model that analyzes the effects of age at circumcision on program impact and cost-effectiveness. The model tracks changes in age-specific MC coverage due to VMMC program circumcisions. Baseline MC prevalence was based on data from the 2011 Demographic and Health Survey. The DMPPT 2 was populated with HIV incidence projections from Spectrum/Goals under an assumption that Mozambique would reach its national targets for HIV treatment and prevention by 2022.

**Results:**

We estimate the VMMC program increased MC coverage among males ages 10 to 49 from 27 percent in 2009 to 48 percent by end of September 2017. Coverage increased primarily in males ages 10 to 29. VMMCs conducted in the national program through the end of September 2017 are projected to avert 67,076 HIV infections from 2010 to 2030. Scaling up circumcisions in males ages 20 to 29 will have the most immediate impact on HIV incidence, while the greatest impact over a 15-year period is obtained by circumcising males ages 15 to 24 in the majority of priority provinces. Circumcising 80 percent of males ages 10 to 29 can achieve 77 percent of the impact through 2030 compared with circumcising 80 percent of males ages 10 to 49.

**Conclusion:**

The VMMC program in Mozambique has made great strides in increasing MC coverage, particularly for males ages 10 to 29. Scaling up and maintaining MC coverage in this age group offers an attainable and cost-effective target for VMMC in Mozambique.

## Introduction

The World Health Organization (WHO) and the Joint United Nations Programme on HIV/AIDS (UNAIDS) identified voluntary medical male circumcision (VMMC) as a priority HIV prevention intervention in areas with high HIV prevalence and low male circumcision (MC) prevalence in 2007 [1]. The Mozambican Ministry of Health (MISAU) subsequently ran a pilot in late 2009 to determine the feasibility of integrating VMMC into the country’s HIV prevention portfolio. The pilot offered VMMC services jointly with HIV testing and counseling, sexually transmitted infection screening and treatment, condom promotion and distribution, and HIV prevention counseling services in five health centers. MISAU eventually expanded the program to 16 additional health centers and circumcised 95,000 men and boys by mid-2012 [2].

Based on the pilot’s success, MISAU developed their 2013–2017 National Male Circumcision Strategy (NMCS) to guide VMMC scale-up efforts in provinces with high HIV incidence and low MC coverage. The NMCS called for circumcising two million males ages 10 to 49 from 2013 to 2017 in the seven provinces with the lowest baseline MC prevalence—Maputo City, Maputo Province, Gaza, Zambezia, Manica, Tete, and Sofala [2]. The targets were selected to reflect rising HIV incidence among youth and impact and cost estimates from a 2011 VMMC modeling study [3]. The study used the original Decision Maker’s Program Planning Tool (DMPPT) to examine the impact and cost of VMMC scale-up in 13 priority countries in eastern and southern Africa with high HIV incidence and low MC coverage, including Mozambique. The study estimated the VMMC program in Mozambique had to circumcise 1,550,000 males to achieve 80 percent MC prevalence among males ages 15 to 49. It also estimated attaining this target by 2015 would avert 220,000 HIV infections between 2011 and 2025, representing 13 percent of expected HIV infections.

By 2015, Mozambique had established VMMC sites in the seven provinces and circumcised 389,000 males. A study estimated that these circumcisions would avert 22,000 HIV infections by 2025 [4]. While boys aged 10 to 14 were easily recruited—constituting about half of all circumcised males—older sexually active men were more difficult to reach. Given VMMC programs across sub-Saharan Africa also had difficulty in reaching older age groups for circumcisions [5–7], the United States President’s Emergency Plan for AIDS Relief (PEPFAR) supported analyses of age-targeting strategies in Malawi, South Africa, Swaziland (now called Eswatini), Tanzania, and Uganda [8–12]. The studies used the Excel-based DMPPT, Version 2 (DMPPT 2) to examine the impact and cost-effectiveness of VMMC scale-up by five-year client age groups and subnational geographies. They determined that in most countries, increasing coverage among males ages 15 to 29 was a cost-effective option that would achieve the most immediate reduction in HIV incidence, as well as the greatest incidence reduction over a 15-year period [13].

The Mozambique VMMC program was interested in a similar assessment of its age-targeting strategy at the province level and desired updated MC coverage estimates at the national, provincial, and district levels to inform program planning and monitoring. This paper describes the application of the DMPPT 2 in 2016 to examine the impact and cost-effectiveness of age-targeting strategies at the provincial level in Mozambique. It also considers the progress and impact of the VMMC program through the end of September 2017.

## Materials and methodology

### Ethics

This study was determined by the Institutional Review Board of the Population Council to be exempt from review on December 15, 2014. The data used in this study included publicly available data and routine program data. All program data were fully anonymized prior to provision to the researchers. No medical records were reviewed as part of this research.

### DMPPT 2 model

The 2.1 version of the DMPPT 2 model used in this exercise is described in detail by Kripke et al. 2016 [4]. DMPPT 2 is a compartmental model implemented in Microsoft Excel 2010 that analyses the effects of age at circumcision on program impact and cost-effectiveness and tracks changes in age-specific MC coverage due to VMMC program circumcisions.

### Data sources

A DMPPT 2.1 model was created for each of the seven VMMC priority provinces in Mozambique (Maputo City, Maputo Province, Gaza, Manica, Sofala, Tete, and Zambezia) using the model inputs available in the supplemental materials (S1 Appendix). The model requires the annual age disaggregated number of VMMCs by district in each priority province to estimate male circumcision coverage by age group and other model outputs. PEPFAR shared this program data by US government fiscal year (FY; October 1 –September 30) with approval from MISAU. Program data for 2015, 2016, and 2017 were provided to the modeling team disaggregated by five-year age group, except for ages 30 to 49. As had been done with data from the other countries, these were disaggregated based on the age distribution of circumcisions conducted in Malawi in PEPFAR FY2013, based on PEPFAR program data. VMMCs for FYs 2010–2014 were disaggregated by age based on the final 2015 age distributions. The percentage age disaggregation applied are available in S1 Table. The number of VMMCs by age group used in each model are in S2 Table for the seven priority provinces. Program VMMCs were reported in the districts where they were performed.

The baseline MC prevalence estimates before the start of the VMMC program were obtained from the 2011 Demographic Health Survey (DHS) [14] for each province. While the VMMC program started in 2009, only 1,032 VMMCs had been conducted by the end of 2010, which would not have significantly affected MC prevalence as measured in the 2011 DHS, so the authors assumed the survey provided reasonable baseline estimates. Because district-level MC prevalence was not available, the provincial MC prevalence was used for each district.

Population size data from Spectrum [15] were based on the World Population Prospects [16], which are projected based on the 2007 Census [17]. Updated age-disaggregated census data are not available, nor have they been incorporated into the World Population Prospects projections.

The VMMC Program and PEPFAR teams advised using the unit cost in Mozambique for VMMC of $93.58 based on an analysis of expenditures reported by implementing partners, as per the standard PEPFAR methodology at the time. This cost includes: personnel, equipment (e.g., procurement and maintenance of VMMC mobile units), consumables, vehicles, training, oversight, and demand creation. Donor costs of administering the program, the cost of space in fixed units, and the cost of overhead (e.g. water, electricity, maintenance, depreciation) were not included in the VMMC unit cost.

DMPPT 2 is populated with annual age-specific HIV incidence and population projections from the Goals module in the Spectrum suite of models [18]. Goals is a dynamic, compartmental model that simulates HIV transmission, morbidity, and mortality consequences in adults ages 15 to 49 based on inputs for demography, HIV epidemiology and disease progression, sexual behavior, and the impact of HIV prevention and treatment interventions. In Spectrum, Goals is linked to the AIDS Impact Model (AIM), which does statistical fitting of HIV sero-prevalence data from antenatal clinic surveillance and national household surveys [18]. The country-validated 2014 Spectrum/AIM and Goals v5.44 files for the South and Central regions were used to populate the DMPPT 2.1 model with population, mortality, and HIV incidence projections. Provincial HIV incidence projections for the provincial DMPPT 2 models were obtained as follows: we exported regional HIV incidence estimates by age from the Spectrum/Goals files, which assume “test and start”—initiating antiretroviral treatment (ART) immediately after HIV infection is confirmed—is implemented starting in 2016. The provincial to regional HIV incidence ratio was calculated with data from the regional Spectrum/AIM files. This incidence ratio scaled the regional HIV incidence by age from Spectrum/Goals for each year between 2009 and 2020. For each year between 2021 and 2050, we scaled regional HIV incidence by the provincial to regional HIV incidence ratio for 2020. Provincial population estimates were obtained by scaling the regional Goals population estimates by the province-to-region population proportion from the 2007 Census. The provincial DMPPT 2 files used the annual age-specific mortality projections from the regional Goals files directly.

### Analytical approaches

The analytical approach to examining the effect of client age on the impact of scaling up VMMC is outlined in other papers in this collection [19, 20]. For each individual five-year and combined age group (e.g., 15 to 19, 15 to 49), we created a scenario in which MC coverage was scaled up linearly from baseline levels in 2016 (VMMC program data were not included as part of the age prioritization analysis, so this analysis assumed scale-up starting in 2016 from the MC prevalence estimates from the 2011 DHS) to 80 percent in 2020, while coverage was maintained constant at baseline levels for other age groups. In each scenario, MC coverage was maintained at 2020 levels for each age group between 2020 and 2050. For each scenario, we compiled and compared annual HIV incidence in the baseline and scale-up scenarios, and the total number of VMMCs required during the scale-up phase (2016 to 2020). Likewise, the model outputs were measured over the 15-year period between 2016 and 2030, inclusive, for each scenario: the total number of HIV infections averted in the population (including secondary infections averted among females, see [19, 20]); the number of VMMCs per HIV infection averted; and the total cost of the VMMC program. These outputs were discounted at a rate of 3 percent per year starting in 2017. The uncertainty of the HIV incidence projections was estimated using the Goals uncertainty analysis and model fitting tool. Estimates were based on the range of possible model parameters that could provide a good fit to the historical HIV prevalence data. The approach is described in detail in S1 Appendix here [13]. Male circumcision uptake rate was calculated by dividing the number of circumcisions in a given age group in a given year by the number of uncircumcised males in that age group and year.

## Results

### Age prioritization

We assessed the impact and cost-effectiveness of age-targeting strategies in each province using the same analytical approach applied in Malawi, South Africa, Swaziland, Tanzania and Uganda. Given the results are similar to those of previous model applications [8–12], we are only presenting a summary of the results here. The figures for each metric and province are available in S2 Appendix. Table 1 lists the male age cohorts with the greatest impact as measured by the following metrics: immediacy of impact (HIV incidence reduction in the first five years), magnitude of impact (HIV incidence reduction over 15 years), efficiency (number of VMMCs per HIV infection averted over 15 years), and cost-effectiveness (cost per HIV infection averted over 15 years).

**Table 1:** Male age cohorts with greatest impact by metric and province.

Parameter	Priority age groups	Province
Immediacy (HIV incidence reduction in the first 5 years)	20–24	All priority provinces
	25–29	Gaza, Sofala, Tete, Zambezia
Magnitude (HIV incidence reduction over 15 years)	15–19, 20–24	All priority provinces
	10–14	Maputo Province, Maputo City, Zambezia
Efficiency (number of VMMCs per HIV infection averted over 15 years)	20–24, 25–29, 30–34	All priority provinces
	15–19	Maputo City, Maputo Province, Zambezia
Cost-effectiveness (cost per HIV infection averted over 15 years)	15–34	All priority provinces

For each province, we compared the projected reductions in HIV incidence in the general population from reaching 80 percent MC coverage in each five-year age group to a scenario in which MC coverage remains unchanged since 2009 (S1 Appendix). In the first five years, the most immediate reduction in HIV incidence would be achieved by scaling-up MCs in males ages 20 to 24 in all priority provinces. By 2030, reaching the target coverage in males ages 15 to 19 and 20 to 24 achieved the greatest reduction in HIV incidence in the priority provinces. The analyses revealed that circumcising males ages 30+ would result in limited HIV incidence reduction in both the short- and long-term. In terms of efficiency, the projections suggest fewer circumcisions are needed to avert an HIV infection when scaling-up MCs in males ages 20 to 24, 25 to 29, and 30 to 34 in the priority provinces (S2 Appendix).

The analyses considering the magnitude, immediacy, and efficiency of impact assumed single five-year age groups were circumcised one at a time. We also examined scenarios in which combined age groups, such as 10 to 49 and 15 to 34, reached 80 percent MC coverage. Scaling-up MCs for the 10 to 49 or 15 to 49 age groups averted the greatest number of HIV infections, over 85,000 infections by the end of 2030 (S2 Appendix). Mozambique can avert 77.0 and 87.8 percent of these HIV infections by targeting a narrower, younger cohort like males ages 10 to 29 or 10 to 34, respectively. In terms of cost-effectiveness, the cost per HIV infection averted over 15 years was lowest when reaching the target coverage among males ages 15 to 34 (S2 Appendix).

### Progress toward numerical targets

**Fig 1:**
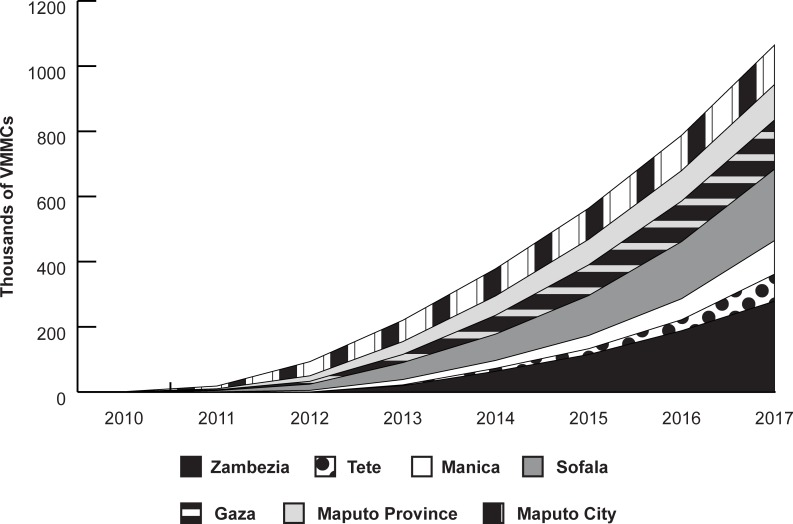
The cumulative number of VMMCs in Mozambique from 2010 to 2017 by province.

The total number of VMMCs performed each US government fiscal year in the seven priority provinces increased from 74,827 in 2012, to 158,264 in 2014, to 277,293 in 2017. By the end of September 2017, the VMMC program had circumcised a total of 1,065,429 males (Fig 1). The greatest numbers of circumcisions were performed in Zambezia and Sofala provinces, which accounted for 26.1 and 20.6 percent of all circumcisions, respectively. Circumcisions in adolescents ages 10 to 19 accounted for 77.6 percent of all circumcisions during this period. By age group, circumcisions in males ages 10 to 14 and 15 to 19 represented 47.1 and 30.5 percent of all circumcisions, respectively. Mozambique circumcised 972,307 males ages 10 to 49 between 1 October 2012 and the end of September 2017, which represents 48.6 percent of the national goal of two million circumcisions by 2017.

### Male circumcision coverage by age group

**Fig 2:**
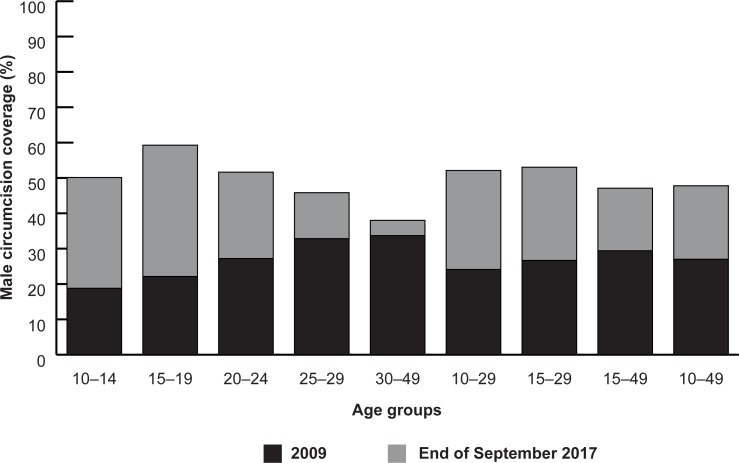
Baseline MC prevalence before the start of the VMMC program in 2009 and modeled coverage estimates by the end of September 2017 across all priority provinces, by select age groups.

We estimated male circumcision coverage at the national, provincial, and district levels by combined and five-year age groups for males ages 10 to 14, 15 to 19, 20 to 24, 25 to 29, 30 to 34, 35 to 39, 40 to 44, and 45 to 49. The combined age groups, males ages 10 to 49 and 15 to 29, were selected based on the age-targeting strategies outlined in the NMCS and in PEPFAR guidance, respectively. Circumcision coverage across all the VMMC priority provinces increased from 27.0 in 2009 to 47.8 percent among males ages 10 to 49 by the end of September 2017 (Fig 2). The greatest absolute and relative increase in coverage was observed in males ages 10 to 14 and 15 to 19 at the national level. We estimate national coverage reached 53.0 percent among males ages 15 to 29 and is highest among males ages 15 to 19 (59.3 percent), 20 to 24 (51.6 percent), and 10 to 14 (50.1 percent).

Our estimates in Table 2 show that Maputo City is the only province that has achieved close to 80 percent coverage among males ages 10 to 49, with a high coverage among males ages 15 to 29 (86 percent). Maputo Province follows closely behind with 71 percent coverage among males ages 15 to 29. (Maputo City and Maputo Province are mutually exclusive geographic and administrative areas within Mozambique.) Zambezia and Gaza have all surpassed 50 percent coverage among males ages 10 to 49. Sofala and Gaza saw the greatest increases in coverage among males ages 10 to 49; coverage for males ages 15 to 19 in Sofala increased from 12 to 71 percent. Gaza has the greatest variability in coverage between adolescent and older males; virtually achieving its target coverage for adolescents ages 10 to 19, while coverage for older men varies between 26 and 50 percent. Tete and Manica are the only provinces that have not achieved more than 50 percent coverage among males ages 15 to 29. These provinces have the lowest baseline circumcision rates, 7 percent for Manica and 2 percent for Tete, and both VMMC programs had a later start than in the other provinces.

**Table 2:** Baseline MC prevalence by age group before start of VMMC program (2009) and modeled coverage estimates by the end of September 2017 (2017).

	MC coverage (%)																	
	10–14		15–19		20–24		25–29		30–49		10–29		15–29		15–49		10–49	
	2009	2017	2009	2017	2009	2017	2009	2017	2009	2017	2009	2017	2009	2017	2009	2017	2009	2017
Zambezia	29	52	35	67	43	64	51	62	51	56	38	61	42	65	45	61	42	59
Tete	1	17	2	21	2	13	2	9	2	3	2	16	2	15	2	11	2	12
Manica	5	25	6	39	7	30	9	20	9	12	7	29	7	31	8	24	7	24
Sofala	11	56	12	71	14	56	17	41	17	25	13	57	14	58	15	45	14	48
Gaza	12	90	15	76	18	50	21	35	22	26	16	66	18	56	20	43	18	52
Maputo Province	31	70	40	73	49	71	58	70	60	64	43	71	48	71	53	68	49	68
Maputo City	39	83	39	88	47	87	56	83	58	68	45	85	47	86	52	78	49	79
All priority provinces	19	50	22	59	27	52	33	46	34	38	24	52	27	53	29	47	27	48

### Programmatic efforts to increase coverage among males ages 15 to 29

**Fig 3:**
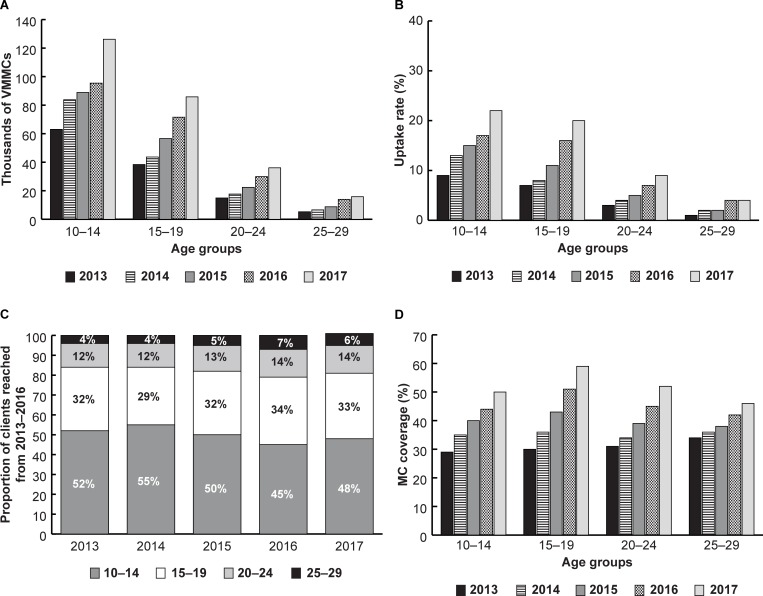
Number of VMMCs (panel a), the uptake rate (panel b), proportion of clients reached (panel c), and MC coverage (panel d) by year and age group from 2013 to end of September 2017 in the priority provinces.

Previous age prioritization analyses in Malawi, South Africa, Swaziland, Tanzania, and Uganda found that circumcising males ages 15 to 29 could increase both the immediacy of impact and cost-effectiveness of VMMC programs. However, as described above, most circumcisions in Mozambique occur in males ages 10 to 19. Like other countries, Mozambique is trying to increase coverage among males ages 20 to 29 in the short term. We assessed the uptake rate of VMMC among males ages 10 to 14, 15 to 19, 20 to 24, and 25 to 29 by US government fiscal year from 1 October 2012 to end of September 2017 using the online version of the DMPPT 2 model [21, 22]. Results are shown in Fig 3.

Fig 3 shows that the annual number of circumcisions and the uptake rate have increased in each age group since 2013. These were highest among males ages 10 to 14 and 15 to 19 years old in every year of program implementation. Mozambique slightly increased the number and proportion of clients ages 15 to 29 in 2016 and 2017 without decreasing the uptake rate or total numbers of circumcisions for males ages 10 to 14. It is worth noting that MC coverage among males ages 20 to 24 surpassed that of males ages 10 to 14 starting in 2016, despite the much lower number of clients and uptake rate in the older age group. This is due to the high proportion of males circumcised in the 10- to 14- and 15- to 19-year-old age groups who age into the 20- to 24-year-old age group.

Trends in the uptake rate vary widely between provinces [not shown]. The uptake rate among clients ages 10 to 14 disproportionately increased compared to clients ages 15 to 29 in Gaza, Maputo Province, and Maputo City. In these provinces, the ratio of younger clients (ages 10 to 14) to older clients (ages 15 to 29) increased between 2013 and 2017. Meanwhile, the uptake rate among clients ages 15 to 19 in Zambezia and Manica surpassed that of clients ages 10 to 14 in 2016 and 2017. However, the ratio of younger clients (ages 10 to 14) to older clients (ages 15 to 29) barely changed.

### Impact of VMMCs conducted through end of September 2017

We assessed the impact of VMMCs from the program start year for each province to 2030 by projecting the number of HIV infections and comparing it to a counterfactual scenario where MC coverage remain unchanged from baseline levels in 2009. Program start dates were: 2010 for Gaza, Maputo City, and Maputo Province; 2011 for Manica and Sofala; and 2013 for Tete and Zambezia. The VMMCs performed through the end of September 2017 in all priority provinces are projected to avert 67,076 HIV infections by 2030; or 15 percent fewer HIV infections compared to a scenario without any program VMMCs (Table 3). The HIV infections averted were not discounted for this part of the analysis. Circumcisions performed in Maputo City, Maputo Province, and Gaza are projected to account for 78.7 percent of the infections averted by VMMC. Fewer HIV infections are projected to be averted in Tete and Manica. We estimated that had Mozambique reached its initial target of 80 percent circumcision coverage among males ages 10 to 49 in the priority provinces by the end of 2017, the VMMC program would have averted 168,593 HIV infections between 2010 and 2030.

**Table 3:** Projected HIV infections averted (IA) by 2030 from program VMMCs conducted through the end of September 2017, total and as a percent of HIV infections in a scenario without any program VMMCs in each province.

Province	IA from program VMMCs by 2030	
	Number	Percent (%)
Zambezia	5,778	11
Tete	480	4
Manica	2,258	6
Sofala	5,603	14
Gaza	18,320	15
Maputo Province	17,893	13
Maputo City	16,744	22
All priority provinces	67,076	15

## Discussion

We applied the DMPPT 2 in Mozambique to study the effect of different age-targeting strategies on the impact and cost-effectiveness of the VMMC program and to estimate the progress and impact of program efforts through 2017. While scale-up efforts fell short of meeting the target of two million circumcisions, the VMMC program reached over half its MC coverage target in males ages 10 to 49 by the end of September 2017. The age prioritization results are in line with similar model findings in other sub-Saharan African countries and support the program’s decision to focus demand generation efforts on males ages 15 to 29 while continuing to accept clients ages 10 to 14 from October 2014 onward. The number of clients and uptake rate for males ages 10 to 29 continued to increase through end of September 2017 without affecting the proportion of older clients ages 20 to 29, despite programmatic efforts to attract more men above age 20. We estimate the circumcisions performed through the end of September 2017 will avert 67,076 HIV infections from 2010 through 2030. The HIV infections averted by the VMMC program represent 40% of the HIV infections that could have been averted had Mozambique reached 80 percent coverage among 10- to 49-year-olds in the priority provinces by the end of September 2017. Notably, our age prioritization results also suggest Mozambique can set their targets to reach a narrower age band such as 10 to 29 and achieve 77.0 percent of the projected impact compared with scaling up to 80 percent coverage among males ages 10 to 49, which may not be programmatically feasible.

Setting targets to achieve coverage among a narrower age band could retain the ambitions of the 2013–2017 NMCS, while ensuring the program is aligned to implementation trends and global guidance on VMMC. The program only came close to reaching its goal of 80 percent coverage among males ages 10 to 49 in Maputo City, the smallest priority province with the most densely concentrated urban population and a higher baseline VMMC coverage across older age groups. These characteristics and a longer scale-up timeline likely helped the program’s relative success there. Overall, however, coverage increased primarily in males under 30 years old even before national VMMC efforts aligned with PEPFAR guidance to increase MC coverage among 10- to 29-year-olds.

The VMMC priority countries in sub-Saharan Africa are struggling to increase MC coverage among 20- to 29-year-olds in response to the modeling results on impact and cost-effectiveness of VMMC by age group [23]. Investments in demand creation to attract clients in this age group have had limited impact, while adolescent clients are much more easily attracted to VMMC services. A recent cluster-randomized controlled trial in Tanzania failed to increase the proportion of clients ages 20 to 29, while increasing the overall VMMC uptake across males ages 10 to 29 [24]. Likewise, our analysis shows efforts across the priority provinces to attract more clients over age 20 have increased VMMC uptake and coverage across age groups without substantially increasing the proportion of clients ages 20 to 29.

These demand generation efforts did increase overall coverage in males ages 15 to 29 without reducing the number of clients ages 10 to 14. Mozambique, like other countries, may wish to consider the medium- and long-term advantages of circumcising young adolescent males. In the medium-term, a study by Njeuhmeli, et al. showed that Mozambique could achieve 86 percent VMMC coverage by the end of 2021 if it aggressively increased VMMC in boys ages 10 to 14, in addition to scaling up efforts for males ages 15 to 29 [25]. In the long-term, circumcising adolescent males will increase coverage among 20- to 29-year-olds as former clients age into the older age groups.

The circumcisions conducted in Mozambique through September 2017 were projected to avert 15 percent of HIV infections by 2030. The number of HIV infections averted will continue to accumulate over time, since VMMC protects men for their entire lives. Nearly 80 percent of these HIV infections averted were projected to be in Maputo City, Maputo Province, and Gaza. While the cumulative number of VMMCs in each of these three provinces was lower than in Zambezia and Sofala, these three provinces had the highest adult male HIV incidence among all of the priority provinces (S1 Appendix). Conversely, Tete and Manica had the lowest projected numbers of HIV infections averted. This is not surprising, given that their VMMC programs scaled up later, the total numbers of VMMCs in Tete and Manica were lower, and the projected HIV incidence in those provinces is also lower. Despite this model projection, program managers in Mozambique intend to continue scaling up VMMC in Tete and Manica, as they believe these provinces are vulnerable to increasing HIV incidence due to high rates of labor migration.

This study has several limitations. The DMPPT 2 requires national and subnational data on population, mortality, HIV incidence and prevalence, and baseline MC. Due to limited subnational data, the model applies provincial HIV incidence/prevalence and baseline MC prevalence to the district level analyses. The population size estimates come from the demographic projections of the 2007 population census, which do not account for recent changes in mortality, fertility, or migration. Changes in these parameters since 2007 could affect both impact estimates and age-specific MC coverage estimates. A new census was conducted in 2017, but age-disaggregated population numbers were not available at the time of this analysis.

Likewise, the VMMC program data does not account for the possibility that clients were circumcised outside their districts of residence. This can affect the coverage estimates in districts to which people travel to obtain VMMC services. For example, migration from suburban districts in Maputo Province to the neighboring Maputo City may distort coverage estimates, as the clients would be counted as contributing to coverage in Maputo City.

The age disaggregation of VMMC clients prior to 2015 was imputed based on age disaggregation of clients in 2015. If the age disaggregation in earlier years of the program was different, that could affect both impact estimates and age-specific coverage estimates. For example, if proportions of older clients were significantly higher prior to 2015, the projected impact of the program would be higher than what is reported here, and the MC coverage in the older age groups would also be higher, while the coverage in the younger age groups would be lower. The age disaggregation of clients aged 30–49 was imputed based on program data from Malawi, which admittedly may not be a reflection of program realities in Mozambique. However, given that so few VMMCs were performed above the age of 30 and that we are not reporting data disaggregated by five-year age group above age 30 in the results of this paper, this limitation does not affect the results presented here.

As for the impact estimates, the HIV incidence estimates upon which they are based have a high degree of uncertainty and are difficult to project into the future, given unknowns related to scale-up of ART, pre-exposure prophylaxis, and other elements of the HIV prevention program. This uncertainty in the HIV incidence estimates leads directly to high levels of uncertainty in the impact estimates.

Another limitation relates to the VMMC unit costs used in the model. VMMC costs were based on analysis of expenditures by experts from MISAU and PEPFAR. Our analysis is only concerned with the relative cost-effectiveness across scenarios, not projecting the actual cost of implementation. However, we did not assume any variation in VMMC unit cost by age group, which could result in different cost-effectiveness results if reaching certain age groups required additional resources [7]. While studies have assessed the cost of VMMC among youth [26], the cost of programs designed to increase the proportion of adult men coming in for circumcision [27], or the variation in VMMC unit costs by program scale, type of facility, and type of staff performing the procedure [28], none have provided direct comparative evidence of how the unit cost of VMMC varies by age. VMMC programs in the region have difficulty reaching men over age 20, and demand creation efforts such as radio spots, billboards, and musical events are directed towards this population. As a simplifying assumption in the cost-effectiveness analysis, costs are assumed to be linearly related to the number of circumcisions performed, although this does not take into account unit cost variations by location, service delivery model, client age group, scale, phase of implementation, and so forth.

In conclusion, the VMMC program in Mozambique has made great strides in increasing MC coverage, particularly for males ages 10 to 29. Its efforts through September 2017 are expected to avert 15 percent of HIV infections through 2030; continued scale-up will avert even more HIV infections. As the country prepares a new VMMC strategy, it may wish to focus its coverage targets on males ages 10 to 29. Scaling up and maintaining MC coverage in males ages 10 to 29 offers an attainable and cost-effective approach to future program efforts in Mozambique.

## Supporting information

S1 Appendix Model inputs by priority province. (XLSX)

S1 Table Age disaggregation of program VMMCs. (DOCX)

S2 Table Number of VMMCs by age, year, and priority province. (DOCX)

S2 Appendix Impact, efficiency, and cost-effectiveness figures from the age prioritization analysis by priority province. (XLSX)
