# Supplementary material for: Age targeting and scale-up of voluntary medical male circumcision in Mozambique
Source: PLoS One. 2019 Feb 22;14(2):e0211958. doi: 10.1371/journal.pone.0211958 (PMC6386365; doi:10.1371/journal.pone.0211958)
Supplement: S1 Table — (DOCX) [file pone.0211958.s002.docx]

Supplemental Table 1: Age disaggregation of Program VMMCs in Mozambique.

| **Age Disaggregation of Program VMMCs** | | | | | | | | |
| --- | --- | --- | --- | --- | --- | --- | --- | --- |
| **2010, 2011, and 2012** | | | | | | | | |
| **EIMC** | As provided to modeling team | | | | | | | |
|  |  |  |  |  |  |  |  |  |
| **1-14 years** | **1-9** | **10-14** |  |  |  |  |  |  |
|  | 0% | 100% |  |  |  |  |  |  |
| **15+** | **15-19** | **20-24** | **25-29** | **30-34** | **35-39** | **40-44** | **45-49** | **50+** |
|  | 58% | 23% | 9% | 4% | 2% | 1.11% | 0.51% | 0.55% |
| **2013 and 2014** | | | | | | | | |
| **EIMC, 1-9, 10-14, 15-19, 20-24, 50+** | As provided to modeling team | | | | | | | |
|  |  |  |  |  |  |  |  |  |
| **25-49** | **25-29** | **30-34** | **35-39** | **40-44** | **45-49** |  |  |  |
|  | 52% | 25% | 14% | 6% | 3% |  |  |  |
| **2015, 2016, and 2017** | | | | | | | | |
| **EIMC, 1-9, 10-14, 15-19, 20-24, 25-29, 50+** | As provided to modeling team | | | | | | | |
|  |  |  |  |  |  |  |  |  |
| **30-49** | **30-34** | **35-39** | **40-44** | **45-49** |  |  |  |  |
|  | 52% | 29% | 13% | 6% |  |  |  |  |

- Program VMMCs for 2010, 2011, and 2012 were provided disaggregated by EIMC, 1-14, and 15+. The modelling team disaggregated the circumcisions for ages 1-14 and 15+ based on the 2015 disaggregation.
- Program VMMCs for 2013 and 2014 were provided disaggregated by EIMC, 1-9, 10-14, 15-19, 20-24, 25-49, and 50+. The modelling team disaggregated the circumcisions for ages 25-49 based on the 2015 disaggregation.
- Program VMMCs for 2015, 2016, and 2017 were provided disaggregated by five-year age groups except for 30-49. The modelling team disaggregated the circumcisions for ages 30-39 based on the 2013 age distribution of circumcisions from PEPFAR Malawi data.
