# Supplementary material for: Age targeting and scale-up of voluntary medical male circumcision in Mozambique
Source: PLoS One. 2019 Feb 22;14(2):e0211958. doi: 10.1371/journal.pone.0211958 (PMC6386365; doi:10.1371/journal.pone.0211958)
Supplement: S2 Table — (DOCX) [file pone.0211958.s003.docx]

Supplemental Table 2A: Number of VMMCs by age and year, Zambezia.

| **Year** | **1–9** | **10–14** | **15–19** | **20–24** | **25–29** | **30–34** | **35–39** | **40–44** | **45–49** | **>50** | **Total** |
| --- | --- | --- | --- | --- | --- | --- | --- | --- | --- | --- | --- |
| **2010** | 0 | 0 | 0 | 0 | 0 | 0 | 0 | 0 | 0 | 0 | 0 |
| **2011** | 0 | 0 | 0 | 0 | 0 | 0 | 0 | 0 | 0 | 0 | 0 |
| **2012** | 0 | 0 | 0 | 0 | 0 | 0 | 0 | 0 | 0 | 0 | 0 |
| **2013** | 0 | 8,621 | 5,944 | 2,768 | 980 | 473 | 264 | 118 | 55 | 80 | 19,303 |
| **2014** | 0 | 19,073 | 13,387 | 6,479 | 2,696 | 1,301 | 726 | 325 | 150 | 71 | 44,212 |
| **2015** | 0 | 16,958 | 19,577 | 8,796 | 2,819 | 1,650 | 920 | 412 | 190 | 120 | 51,443 |
| **2016** | 0 | 22,940 | 28,068 | 12,168 | 5,018 | 2,413 | 1,346 | 603 | 278 | 181 | 73,015 |
| **2017** | 0 | 38,009 | 31,420 | 12,107 | 4,875 | 2,162 | 1,206 | 540 | 249 | 38,009 | 90,568 |

Source: Mozambique national program data

Supplemental Table 2B: Number of VMMCs by age and year, Tete.

| **Year** | **1–9** | **10–14** | **15–19** | **20–24** | **25–29** | **30–34** | **35–39** | **40–44** | **45–49** | **>50** | **Total** |
| --- | --- | --- | --- | --- | --- | --- | --- | --- | --- | --- | --- |
| **2010** | 0 | 0 | 0 | 0 | 0 | 0 | 0 | 0 | 0 | 0 | 0 |
| **2011** | 0 | 0 | 0 | 0 | 0 | 0 | 0 | 0 | 0 | 0 | 0 |
| **2012** | 0 | 0 | 0 | 0 | 0 | 0 | 0 | 0 | 0 | 0 | 0 |
| **2013** | 0 | 276 | 1,360 | 590 | 138 | 67 | 37 | 17 | 8 | 6 | 2,500 |
| **2014** | 0 | 2,706 | 2,373 | 928 | 324 | 158 | 88 | 39 | 18 | 6 | 6,643 |
| **2015** | 0 | 4,045 | 3,040 | 904 | 392 | 126 | 70 | 31 | 15 | 17 | 8,640 |
| **2016** | 0 | 7,958 | 4,601 | 1,422 | 964 | 530 | 296 | 133 | 61 | 29 | 15,994 |
| **2017** | 0 | 17,046 | 18,234 | 7,673 | 3,986 | 1,135 | 633 | 284 | 131 | 32 | 49,154 |

Source: Mozambique national program data

Supplemental Table 2C: Number of VMMCs by age and year, Manica.

| **Year** | **1–9** | **10–14** | **15–19** | **20–24** | **25–29** | **30–34** | **35–39** | **40–44** | **45–49** | **>50** | **Total** |
| --- | --- | --- | --- | --- | --- | --- | --- | --- | --- | --- | --- |
| **2010** | 0 | 0 | 0 | 0 | 0 | 0 | 0 | 0 | 0 | 0 | 0 |
| **2011** | 0 | 113 | 195 | 78 | 30 | 15 | 8 | 4 | 2 | 1 | 448 |
| **2012** | 0 | 1,110 | 1,984 | 797 | 313 | 151 | 84 | 38 | 17 | 18 | 4,514 |
| **2013** | 0 | 3,583 | 4,662 | 2,042 | 589 | 284 | 159 | 71 | 33 | 19 | 11,443 |
| **2014** | 0 | 2,602 | 3,094 | 1,514 | 316 | 153 | 85 | 38 | 18 | 6 | 7,826 |
| **2015** | 0 | 5,791 | 6,463 | 2,786 | 868 | 213 | 119 | 53 | 25 | 29 | 16,347 |
| **2016** | 0 | 9,391 | 9,127 | 3,076 | 1,319 | 470 | 262 | 117 | 54 | 56 | 23,872 |
| **2017** | 0 | 12,977 | 15,785 | 6,750 | 2,211 | 869 | 485 | 217 | 100 | 59 | 39,453 |

Source: Mozambique national program data

Supplemental Table 2D: Number of VMMCs by age and year, Sofala.

| **Year** | **1–9** | **10–14** | **15–19** | **20–24** | **25–29** | **30–34** | **35–39** | **40–44** | **45–49** | **>50** | **Total** |
| --- | --- | --- | --- | --- | --- | --- | --- | --- | --- | --- | --- |
| **2010** | 0 | 0 | 0 | 0 | 0 | 0 | 0 | 0 | 0 | 0 | 0 |
| **2011** | 0 | 535 | 2,036 | 818 | 321 | 155 | 86 | 39 | 18 | 19 | 4,027 |
| **2012** | 0 | 5,983 | 5,271 | 2,118 | 832 | 401 | 224 | 100 | 46 | 50 | 15,024 |
| **2013** | 0 | 16,624 | 9,552 | 3,985 | 1,385 | 667 | 372 | 167 | 77 | 75 | 32,904 |
| **2014** | 0 | 16,309 | 6,883 | 3,398 | 1,177 | 567 | 316 | 142 | 65 | 25 | 28,882 |
| **2015** | 0 | 20,198 | 12,904 | 4,949 | 2,022 | 867 | 483 | 217 | 100 | 58 | 41,798 |
| **2016** | 0 | 17,922 | 20,056 | 8,412 | 3,736 | 1,235 | 689 | 309 | 143 | 96 | 52,597 |
| **2017** | 0 | 20,988 | 13,547 | 5,753 | 2,226 | 738 | 412 | 185 | 85 | 0 | 43,934 |

Source: Mozambique national program data

Supplemental Table 2E: Number of VMMCs by age and year, Gaza.

| **Year** | **1–9** | **10–14** | **15–19** | **20–24** | **25–29** | **30–34** | **35–39** | **40–44** | **45–49** | **>50** | **Total** |
| --- | --- | --- | --- | --- | --- | --- | --- | --- | --- | --- | --- |
| **2010** | 1 | 122 | 129 | 52 | 20 | 10 | 5 | 2 | 1 | 1 | 345 |
| **2011** | 0 | 830 | 536 | 215 | 85 | 41 | 23 | 10 | 5 | 5 | 1,749 |
| **2012** | 0 | 3,795 | 1,675 | 673 | 264 | 127 | 71 | 32 | 15 | 16 | 6,668 |
| **2013** | 0 | 8,127 | 5,797 | 1,480 | 404 | 195 | 109 | 49 | 22 | 27 | 16,210 |
| **2014** | 0 | 18,928 | 10,298 | 2,095 | 752 | 362 | 202 | 91 | 42 | 78 | 32,847 |
| **2015** | 0 | 24,567 | 9,096 | 1,492 | 565 | 259 | 144 | 65 | 30 | 70 | 36,288 |
| **2016** | 0 | 21,400 | 5,650 | 1,393 | 748 | 467 | 260 | 117 | 54 | 153 | 30,242 |
| **2017** | 0 | 19,590 | 4,008 | 1,258 | 717 | 531 | 296 | 133 | 61 | 0 | 26,594 |

Source: Mozambique national program data

Supplemental Table 2F: Number of VMMCs by age and year, Maputo Province.

| **Year** | **1–9** | **10–14** | **15–19** | **20–24** | **25–29** | **30–34** | **35–39** | **40–44** | **45–49** | **>50** | **Total** |
| --- | --- | --- | --- | --- | --- | --- | --- | --- | --- | --- | --- |
| **2010** | 0 | 52 | 289 | 116 | 46 | 22 | 12 | 5 | 3 | 3 | 547 |
| **2011** | 0 | 1,376 | 764 | 307 | 121 | 58 | 32 | 15 | 7 | 7 | 2,686 |
| **2012** | 0 | 7,447 | 3,649 | 1,466 | 576 | 277 | 155 | 69 | 32 | 34 | 13,706 |
| **2013** | 0 | 14,357 | 5,626 | 1,446 | 525 | 253 | 141 | 63 | 29 | 49 | 22,490 |
| **2014** | 0 | 13,000 | 4,458 | 1,529 | 564 | 272 | 151 | 68 | 31 | 71 | 20,144 |
| **2015** | 0 | 11,438 | 3,640 | 1,677 | 915 | 503 | 281 | 126 | 58 | 137 | 18,775 |
| **2016** | 0 | 9,439 | 2,198 | 1,451 | 868 | 532 | 297 | 133 | 61 | 199 | 15,179 |
| **2017** | 0 | 10,904 | 1,375 | 1,083 | 752 | 604 | 337 | 151 | 70 | 108 | 15,383 |

Source: Mozambique national program data

Supplemental Table 2G: Number of VMMCs by age and year, Maputo City.

| **Year** | **1–9** | **10–14** | **15–19** | **20–24** | **25–29** | **30–34** | **35–39** | **40–44** | **45–49** | **>50** | **Total** |
| --- | --- | --- | --- | --- | --- | --- | --- | --- | --- | --- | --- |
| **2010** | 0 | 55 | 50 | 20 | 8 | 4 | 2 | 1 | 0 | 0 | 140 |
| **2011** | 0 | 3,212 | 2,997 | 1,204 | 473 | 228 | 127 | 57 | 26 | 28 | 8,353 |
| **2012** | 0 | 19,324 | 9,090 | 3,652 | 1,434 | 691 | 385 | 173 | 80 | 85 | 34,915 |
| **2013** | 0 | 11,450 | 5,449 | 2,669 | 1,230 | 593 | 331 | 148 | 68 | 125 | 22,063 |
| **2014** | 0 | 11,168 | 3,177 | 1,743 | 800 | 385 | 215 | 96 | 44 | 81 | 17,710 |
| **2015** | 0 | 5,892 | 1,911 | 1,796 | 1,224 | 660 | 368 | 165 | 76 | 100 | 12,192 |
| **2016** | 0 | 6,416 | 1,910 | 1,977 | 1,380 | 848 | 473 | 212 | 98 | 142 | 13,455 |
| **2017** | 0 | 6,727 | 1,513 | 1,469 | 1,108 | 723 | 403 | 181 | 83 | 0 | 12,207 |

Source: Mozambique national program data
